# Reproductive Traits Revealing the Invasion and Coexistence of Two Tilapia Species in the Jinghong Reservoir of the Lower Lancang River, Southwest China

**DOI:** 10.3390/ani16132055

**Published:** 2026-07-03

**Authors:** Ziheng Hu, Liwen Dong, Ke Li, Dongdong Zhai, Yuanyuan Chen, Hongyan Liu, Fei Xiong, Xinbin Duan, Mingdian Liu

**Affiliations:** 1 Hubei Engineering Research Center for Protection and Utilization of Special Biological Resources in the Hanjiang River Basin, School of Life Sciences, Jianghan University, Wuhan 430056, China; hzh01f@163.com (Z.H.);; 2 Hubei Key Laboratory of Environmental and Health Effects of Persistent Toxic Substances, Jianghan University, Wuhan 430056, China; 3 National Agricultural Science Observing and Experimental Station of Chongqing, Yangtze River Fisheries Research Institute, Chinese Academy of Fishery Science, Wuhan 430223, China

**Keywords:** *Coptodon zillii*, *Oreochromis niloticus*, reproductive strategy, biological invasion

## Abstract

*Coptodon zillii* and *Oreochromis niloticus* are dominant invasive fish species that have successfully established and coexisted in the Jinghong Reservoir of the lower Lancang River, Southwest China. To explore the mechanisms underlying their invasion and coexistence from the perspective of reproductive traits, we conducted monthly sampling from January to December 2025 to compare key reproductive parameters, including size at first sexual maturity, sex ratio, breeding season, spawning pattern and fecundity. The results showed that the predicted sizes at first sexual maturity for females and males of *C. zillii* (83.4 mm and 81.7 mm, respectively) were significantly smaller than those of *O. niloticus* (127.7 mm and 125.8 mm, respectively). Both species exhibited early sexual maturation. The sex ratio of *C. zillii* was approximately 1:1, whereas that of *O. niloticus* was significantly female-biased. The breeding season of *C. zillii* lasted from April to September, peaking in May and June, while *O. niloticus* spawned from May to November, with a peak in August, showing a staggered temporal distribution. Both species were batch spawners; however, *O. niloticus* had significantly larger egg diameters than *C. zillii*. Absolute fecundity, length-relative fecundity and weight-relative fecundity were significantly higher in *C. zillii* than in *O. niloticus*. Moreover, the absolute fecundity of *C. zillii* showed stronger correlations with body length, body weight, net weight and gonadal weight. *C. zillii* adopted a more r-selected strategy, while *O. niloticus* exhibited a more K-selected strategy. The two species displayed clear divergence in their reproductive strategies, including size at maturity, breeding season, egg diameter and fecundity, which reduced interspecific competition and promoted niche separation and coexistence in the Jinghong Reservoir.

## Simple Summary

Two invasive fish species, *Coptodon zillii* and *Oreochromis niloticus*, have successfully invaded and coexisted in the Jinghong Reservoir of the lower Lancang River, Southwest China. To understand their successful invasion and coexistence, we compared the key reproductive traits. The results showed that both species exhibited reproductive traits conducive to invasion, including early sexual maturation, prolonged breeding seasons and batch spawning. Meanwhile, the two species showed clear divergence in their reproductive strategies: *C. zillii* followed a more r-selected strategy, whereas *O. niloticus* tended toward a more K-selected strategy. Additionally, temporal staggering of their breeding seasons effectively reduced direct competition for reproductive resources.

## 1. Introduction

Biological invasion, a major driver of biodiversity loss and a serious threat to riverine ecosystems [1], is particularly prominent in the lower Lancang River. Originating from the northern foothills of the Tanggula Mountains, the Lancang River is an international river that flows from north to south through China, Myanmar, Laos, Thailand, Cambodia and Vietnam. The section within China is called the Lancang River, while the section beyond China’s borders is known as the Mekong River. The lower reach of the Lancang River, extending from Jinglin Bridge to the estuary of Nanla River, is characterized by a large elevational gradient (914–465 m) and complex climate, which support rich freshwater fish diversity [2]. However, anthropogenic disturbances have intensified markedly over recent decades, particularly from hydropower development [3,4]. Since 1993, eleven cascade hydropower stations have been constructed and are now operational along the mainstream of the Lancang River [5]. Previous studies have shown a significant increase in the number of non-native fish species in the lower reaches following cascade hydropower development. Hong et al. [6] reported that the number of non-native fish species in the mainstream of the lower Lancang River increased from one species before 1990 to 22 species during 2008–2013, and further to 37 species during 2018–2019. Similarly, Fu et al. [7] surveyed the Xishuangbanna section of the lower Lancang River and found that the number of non-native fish species rose from six in 1990 to 17 in 2013, reaching 32 by 2020. The introduction of non-native fishes has severely compressed the living space of native species and has become a key stressor driving biodiversity decline in this region. Among these introduced species, *C. zillii* and *O. niloticus* have exhibited strong adaptability and expansive capacity, and now they have formed dominant populations in multiple reservoirs of the Lancang River, presenting a pattern of invasion and coexistence.

*C. zillii* and *O. niloticus* belong to the family Cichlidae. Both species are native to Africa and the Middle East and were introduced into China from Thailand in 1978 for aquaculture [8,9]. Due to its small body size, *C. zillii* was abandoned by aquaculturists, subsequently escaped into natural waters, and has since established stable populations across various aquatic systems in southern China. It has been listed in the Key Managed Invasive Alien Species Catalog by the Ministry of Agriculture and Rural Affairs [10,11]. In contrast, *O. niloticus*, characterized by fast growth and a larger body size, has become the dominant species in aquaculture and likely spread widely into natural waters through flood escapes [12]. Currently, both species exhibit significant competitive advantages in the rivers and lakes across southern and southwestern China [11,13]. The coexistence of two or even multiple tilapia species invading the same natural waters is a common phenomenon. However, studies on the mechanisms by which tilapia species invade and coexist in the same habitat remain scarce.

The plasticity and divergence of reproductive strategies were key factors determining the success of colonization, expansion potential and interspecific competitive patterns of introduced species in novel environments [14]. Invasive species, such as *Clarias gariepinus* in China [15], *Perca fluviatilis* in the Murray River of Australia [16] and *Alosa sapidissima* in North America [17], have demonstrated strong population expansion capabilities due to their prolonged breeding seasons and high fecundity. For coexisting invasive species, divergence in reproductive strategies might promote coexistence by reducing the intensity of interspecific competition [18]. For example, Guo et al. [19] studied two coexisting invasive goby species in Lake Erhai and revealed significant differentiation in spawning time, size at sexual maturity and fecundity. Currently, although *C. zillii* and *O. niloticus* have established a pattern of invasion and coexistence in the downstream reservoir zone of the Lancang River, the key reproductive traits and reproductive strategies of the two species in this region remain poorly understood.

This study focused on *C. zillii* and *O. niloticus*, which have successfully established and coexisted in the Jinghong Reservoir in the lower Lancang River, and conducted a systematic comparison of their reproductive traits. By analyzing the key parameters, including the size at first sexual maturity, sex ratio, breeding season, spawning pattern and fecundity, we aim to clarify: (1) the reproductive strategies of the two invasive tilapia species and their differentiation; (2) the possible mechanisms underlying their successful invasion and coexistence in the Jinghong Reservoir of the Lancang River.

## 2. Materials and Methods

### 2.1. Study Area

The Jinghong Reservoir, located in Xishuangbanna, Yunnan Province, is a large canyon-type reservoir formed by the impoundment of the Jinghong Hydropower Station on the lower Lancang River. The reservoir began impounding water in April 2008, stretching approximately 108 km in length, with a maximum dam height of 108 m, a normal water level of 602 m and a total storage capacity of 11.39 billion m^3^. Since impoundment, the original riverine ecological structure and function have been disrupted. Comparable evidence from the upper Mekong showed that dam-induced changes in flow and thermal regimes could disrupt fish recruitment [20]. This region lies at the northern margin of the tropics, characterized by a warm, humid climate and abundant food resources, providing favorable conditions for the establishment and invasion of non-native fishes [21,22]. From 2023 to 2025, our research team conducted a fish survey in the Jinghong Reservoir and recorded a total of 15 non-native fish species. Among these, *C. zillii* and *O. niloticus* have become the dominant species in the reservoir. Together, these two tilapia species accounted for 28.45% of the total fish abundance (*C. zillii*: 15.59%; *O. niloticus*: 12.86%) and 50.10% of the total fish biomass (*C. zillii*: 21.72%; *O. niloticus*: 28.38%), presenting a pattern of invasion and coexistence.

### 2.2. Fish Survey and Sampling

**Figure 1:**
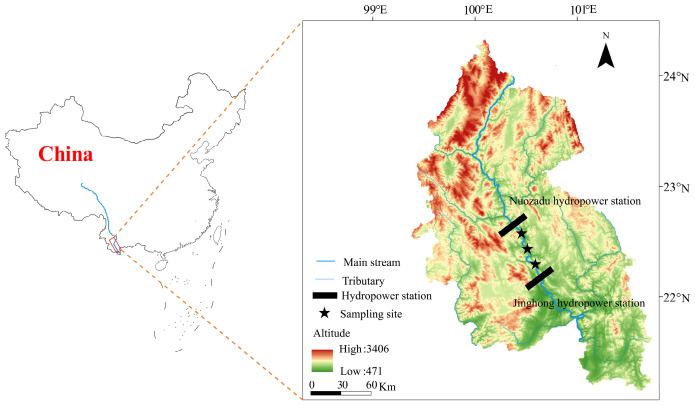
Sampling sites of tilapia in the Jinghong Reservoir, Lancang River. Map source: http://bzdt.ch.mnr.gov.cn/index.html, accessed on 20 May 2025.

From January to December 2025, specimens of the two tilapia species were collected monthly from the Jinghong Reservoir, covering the head, middle and tail sections of the reservoir (Figure 1). Sampling gear consisted of gill nets (one surface net and one bottom net) and two ground cages. Each gill net was assembled from four panels with different mesh sizes (2.0 cm, 6.0 cm, 10.0 cm and 14.0 cm). Each mesh panel measured 50 m in length and 2 m in height; each composite gill net had a total length of 200 m. The ground cage had a mesh size of 0.8 cm and measured 18 m in length, 0.45 m in width and 0.33 m in height. The type and amount of sampling gear remained consistent across all sampling events. A total of 502 *C. zillii* were collected from the Jinghong Reservoir of the Lancang River, consisting of 264 females, 222 males and 16 individuals of unidentified sex. Additionally, 505 *O. niloticus* were collected, comprising 328 females, 163 males and 14 individuals of unidentified sex.

All specimens were measured in a fresh state for body length (*L*), body weight (*W*), net weight (*W_n_*) (body weight after removal of all internal organs) and gonadal weight (*W_g_*). Body length was recorded using a fish measuring board (precision: 1 mm), and weights were measured using a BW3200 electronic balance (precision: 0.1 g). Sex identification was determined based on gonadal differences after dissection for individuals at Stage II and above. Gonadal development was classified into six stages (I–VI) following the method of Ding et al. [23]. Gonads were preserved in 10% formalin solution and transported to the laboratory for egg diameter and fecundity analysis.

### 2.3. Reproductive Biology Indicators

Gonadosomatic index: The degree of gonadal development in females was assessed using the gonadosomatic index (GSI) [24], calculated as

GSI = (*W_g_*/*W_n_*) × 100%

where *W_g_* is the gonadal weight (g) and *W_n_* is the net weight (g).

Size at first sexual maturity: The size at first sexual maturity was predicted using the *L*_50_ method, defined as the body length at which 50% of individuals in the population reached sexual maturity [25]. Fish were grouped into 10 mm body length intervals, and the proportion of mature individuals within each interval was calculated as the maturity proportion for that length group. Logistic regression was used to model the relationship between the proportion of mature individuals and body length. The logistic equation was as follows:

$$P=1/[1+e^{-k(L_{mid}-L_{50})}]$$

where P is the proportion of mature individuals in each length group (ranging from 0 to 1), *k* is the slope of the logistic curve, *L_mid_* is the midpoint of the length interval (mm), and *L*_50_ is the mean length at first sexual maturity (mm).

Spawning pattern: Ovaries from 10 individuals with Stage IV gonadal maturity were randomly selected for egg diameter distribution analysis. From each ovary, 0.1 g of eggs was taken from the anterior, middle and posterior portions. After being dispersed, 300 mature eggs were randomly selected from each portion for measurement. The major and minor diameters of each egg were measured under a microscope (Olympus China Co., Ltd., Shanghai, China) using Lightools 7.3 photoelectric image analysis software, and the mean value was defined as the egg diameter (Ed, mm). Spawning pattern was determined based on the frequency distribution of egg diameters. The presence of multiple distinct peaks in the egg diameter distribution indicated a batch-spawning pattern.

Fecundity estimation: Individual fecundity was used to assess the reproductive potential of the fish. Ovaries were collected from 46 *C. zillii* and 50 *O. niloticus* specimens at gonadal maturity Stage IV. For each ovary, approximately 1 g (*W_s_*) of ovarian tissue was randomly taken from the anterior, middle and posterior portions for egg counting [26]. Absolute fecundity (*F*), length-relative fecundity (*F_L_*) and weight-relative fecundity (*F_W_*) were calculated using the following formulas, respectively:

*F* = *N* × *W_g_*/*W_s_* *F_L_* = *F*/*L* *F_w_* = *F*/*W_n_*

where *F* is the individual absolute fecundity (number of eggs), *N* is the number of eggs counted, *W_g_* is the gonadal weight (g), *W_s_* is the subsample weight (1 g), *F_L_* is the length-relative fecundity (eggs/mm), *L* is the body length (mm), *F_W_* is the weight-relative fecundity (eggs/g) and *W_n_* is the net weight (g).

### 2.4. Statistical Analysis

The Mann–Whitney *U* test was used to examine differences between body length and body weight between the two tilapia species (interspecific) and between sexes within each species (intraspecific). The chi-square (χ^2^) test was used to evaluate whether sex ratios deviated significantly from 1:1 and whether there was a significant difference in sex ratio between the two species. Welch’s *t*-test was applied to compare egg diameters between the two species. Regression models were fitted to establish the relationships between absolute fecundity and body length, body weight, net weight and gonadal weight. All data were processed and analyzed using Microsoft Excel 2021 and R software (version 4.5.1).

## 3. Results

### 3.1. Body Length and Weight Distribution

**Figure 2:**
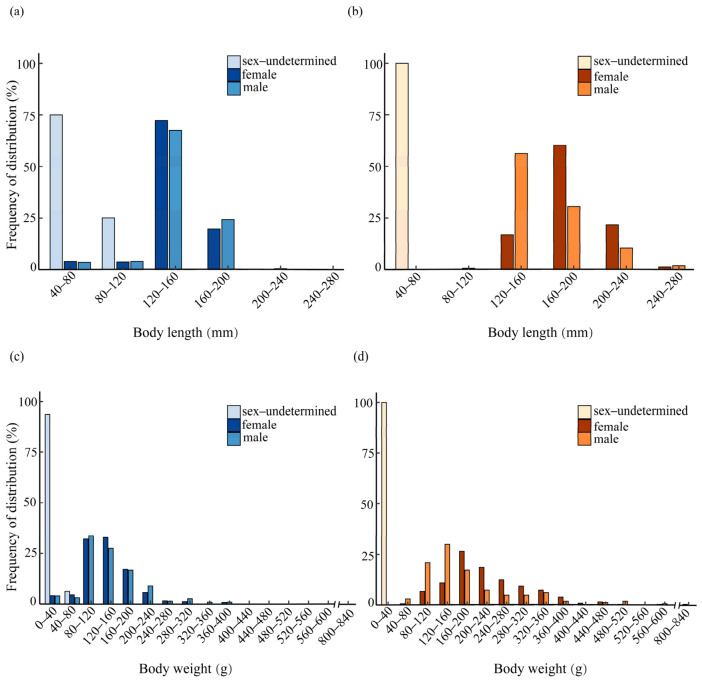
Distribution of body length and weight of *C. zillii* (**a**,**c**) and *O. niloticus* (**b**,**d**).

The body length and body weight data for the two tilapia species are presented in Table 1, and their frequency distributions are shown in Figure 2. For *C. zillii*, the females ranged from 53 to 200 mm in body length and from 6.4 to 388.7 g in body weight; the males ranged from 52 to 213 mm in body length and from 5.4 to 399.4 g in body weight. Both sexes of *C. zillii* showed the highest proportion of individuals in the 120–160 mm length range (72.35% for females and 67.57% for males) and in the 80–160 g weight range (65.14% for females and 61.26% for males). For *O. niloticus*, the females ranged from 127 to 280 mm in body length and from 74.3 to 815 g in body weight; the males ranged from 120 to 250 mm in body length and from 67.3 to 591.5 g in body weight. Female *O. niloticus* were most abundant in the 160–200 mm length range (60.37%), whereas males were most abundant in the 120–160 mm range (56.44%). Females were most abundant in the 160–200 g range (26.53%), while males were most abundant in the 120–160 g range (30.06%).

Interspecific comparisons using the Mann–Whitney *U* test revealed that both females and males of *O. niloticus* were significantly larger in body length and body weight than those of *C. zillii* (females: length *U* = 9579.50, weight *U* = 13,336.50, both *p* < 0.001; males: length *U* = 12,102.50, weight *U* = 13,506.50, both *p* < 0.001). Intraspecific analysis showed no significant differences in body length and weight between sexes for *C. zillii* (length *U* = 28,246.00, weight *U* = 27,442.50, both *p* > 0.05). In contrast, for *O. niloticus*, females were significantly larger than males in both body length and weight (length *U* = 38,399.00, weight *U* = 36,754.50, both *p* < 0.001).

### 3.2. Size at First Sexual Maturity

**Figure 3:**
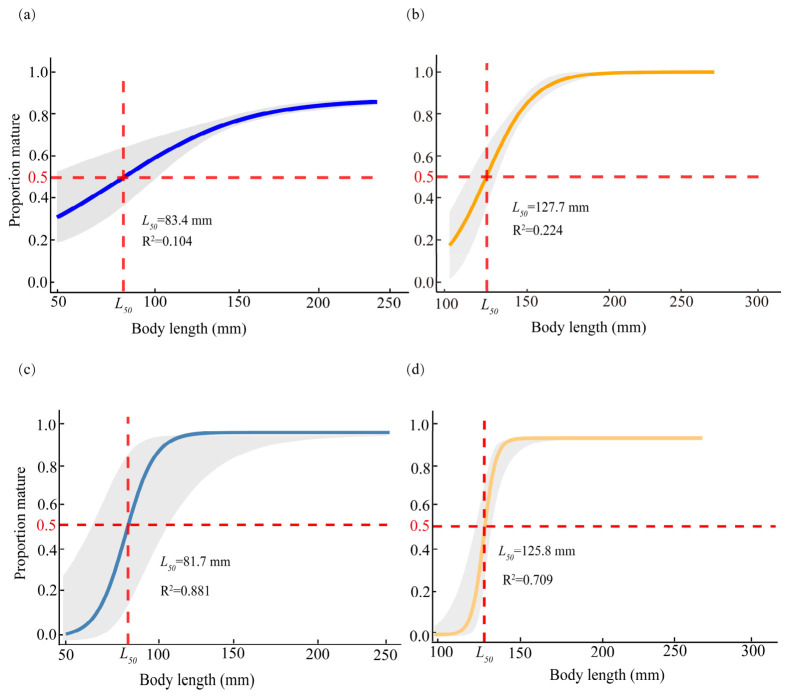
Size at first sexual maturity (*L*_50_) was predicted for female and male *C. zillii* (**a**,**c**) and *O. niloticus* (**b**,**d**). The grey area represented the 95% confidence interval.

The relationship between body length and the proportion of mature individuals was fitted using a logistic equation (Figure 3). The size at first sexual maturity (*L*_50_) for female and male *C. zillii* was predicted at 83.4 mm and 81.7 mm, respectively, which values were significantly smaller than those for *O. niloticus* (127.7 mm and 125.8 mm, respectively), indicating that *O. niloticus* reached sexual maturity at a larger body size.

### 3.3. Sex Ratio

The overall female-to-male sex ratio of *C. zillii* was 1.19:1, which did not deviate significantly from 1:1 (χ^2^ = 3.63, *df* = 1, *p* > 0.05). In contrast, the overall sex ratio of *O. niloticus* was 2.01:1, showing a significant female bias and deviating markedly from 1:1 (χ^2^ = 55.50, *df* = 1, *p* < 0.01). The sex ratios differed significantly between the two species (χ^2^ = 15.94, *df* = 1, *p* < 0.01). The sex ratio of *C. zillii* maintained a relatively stable sex ratio close to 1:1 throughout most months of the year. Conversely, *O. niloticus* exhibited pronounced seasonal fluctuations, with the highest proportion of females occurring in February and March, declining from April to June, rising again from July to October, and decreasing in November and December (Table 2).

### 3.4. Breeding Season

**Figure 4:**
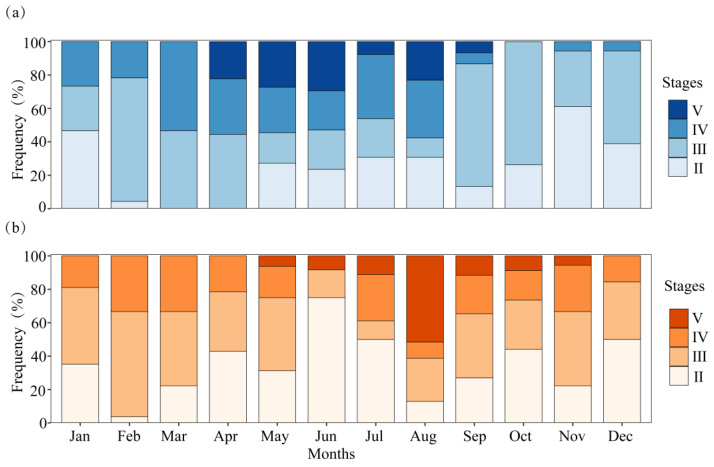
Monthly variation in the proportion of female gonadal developmental stages for *C. zillii* (**a**) and *O. niloticus* (**b**).

**Figure 5:**
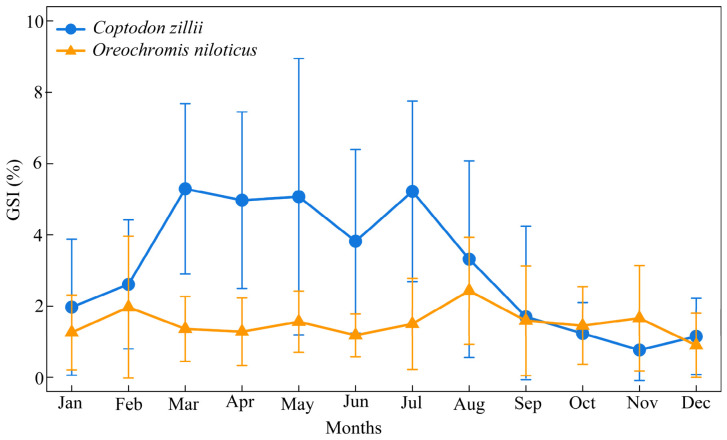
Monthly variation in the gonadosomatic index (GSI) of female *C. zillii* and *O. niloticus*.

In *C. zillii*, ovaries were predominantly at Stages II and III during January and February, with a rapid increase in the proportion of Stage IV in March. Stage V ovaries first appeared in April, marking the onset of the spawning season. The spawning peak occurred in May and June, with Stage V ovaries accounting for 27.27% and 29.41%, respectively. Spawning ceased in October, with only Stage II and III ovaries observed. Although Stage IV ovaries occurred sporadically in November and December, their proportions were very low. In contrast, Stage V ovaries of *O. niloticus* first appeared in May, with a spawning season extending from May to November and a peak in August, when Stage V ovaries accounted for 51.61% (Figure 4). The trend in the gonadosomatic index (GSI) was generally consistent with the pattern of ovarian developmental stages, and the overall GSI of *O. niloticus* was lower than that of *C. zillii* (Figure 5).

### 3.5. Spawning Pattern

**Figure 6:**
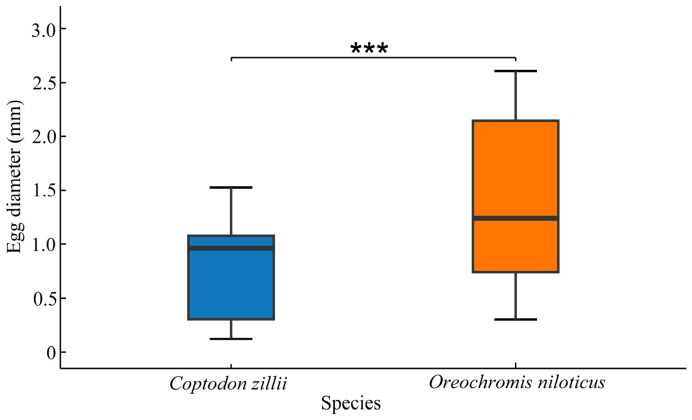
Comparison of Stage IV egg diameters of *C. zillii* and *O. niloticus.* *** indicates extremely significant difference.

**Figure 7:**
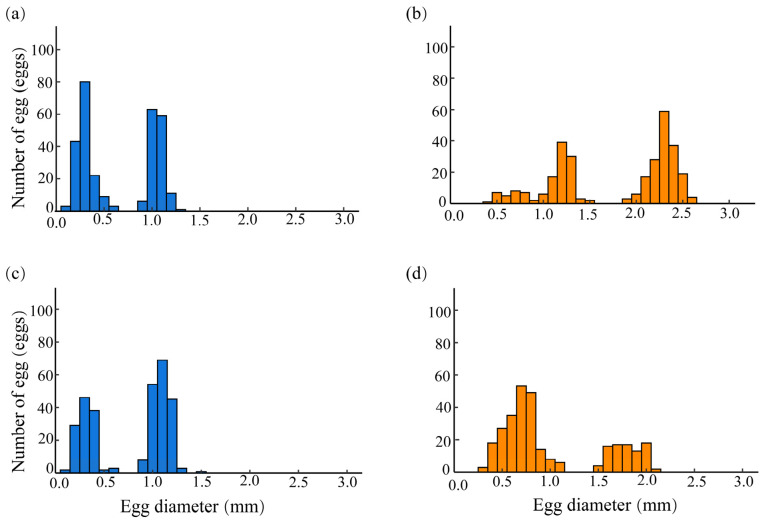
Distribution of Stage IV egg diameters for two *C. zillii* (**a**,**c**) and two *O. niloticus* (**b**,**d**) samples.

The egg diameters ranged from 0.12 to 1.53 mm in *C. zillii* (mean ± SD: 0.72 ± 0.39 mm) and from 0.30 to 2.61 mm in *O. niloticus* (mean ± SD: 1.40 ± 0.70 mm). The egg diameter of *O. niloticus* was significantly larger than that of *C. zillii* (Welch’s *t*-test, *p* < 0.001) (Figure 6). The frequency distribution of egg diameters (Figure 7) showed two distinct peaks for both species, indicating that both *C. zillii* and *O. niloticus* were batch spawners.

### 3.6. Fecundity

The fecundity parameters for both species are presented in Table 3. Although sexually mature *O. niloticus* had a significantly greater body length and body weight than *C. zillii* (*t*-test, *p* < 0.01), all fecundity parameters (*F*, *F_L_* and *F_W_*) were significantly higher in *C. zillii* than in *O. niloticus* (*t*-test, *p* < 0.01), indicating that *C. zillii* has a greater reproductive output.

**Figure 8:**
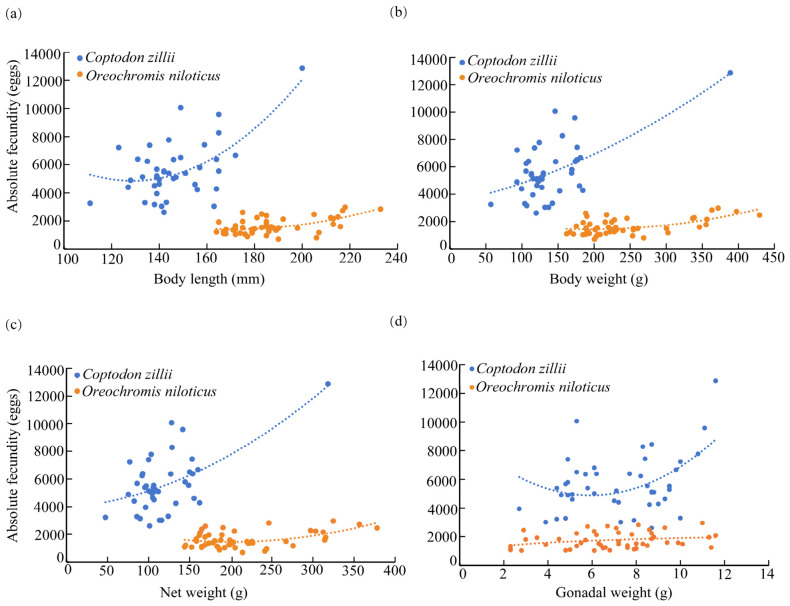
Absolute fecundity of *C. zillii* and *O. niloticus* in relation to body length (**a**), body weight (**b**), net weight (**c**) and gonadal weight (**d**).

Regression analyses (Figure 8) showed that absolute fecundity was significantly positively correlated with body length, body weight, net weight and gonadal weight in both species (*p* < 0.05), with stronger correlations observed in *C. zillii*.

## 4. Discussion

### 4.1. Invasive Reproductive Traits of the Two Tilapia Species

The size at first sexual maturity directly determines the population development potential of fish [27,28]. In this study, the sizes at first sexual maturity for female and male *C. zillii* were predicted to be 83.4 mm and 81.7 mm, respectively, and those for *O. niloticus* were estimated at 127.7 mm and 125.8 mm, respectively. Both species reached sexual maturity at small body sizes, which might enable them to enter the reproductive phase earlier, thereby driving rapid population expansion and facilitating successful invasion. Compared with other populations, *C. zillii* in the Jinghong Reservoir matured at smaller sizes than those in the Shuikou Reservoir and Guangxi, China [29,30]. *O. niloticus* matured at smaller sizes than those in the Guangxi population of China [30] and the Lake Victoria population of Tanzania [31]. Similar early sexual maturation, the trait that favored rapid colonization of new habitats, has been reported in various invasive fish species [32,33].

The sex ratio in tilapia was closely related to their reproductive strategy [34]. In inland waters of Guangxi, China, the sex ratios of both invasive *C. zillii* and *O. niloticus* were approximately 1:1 [30], whereas in the Shanmei Reservoir of Fujian, the sex ratio of invasive *C. zillii* deviated significantly from 1:1 [35]. Russell et al. [36] suggested that tilapia sex ratios varied with species and geographical location, and that such variation benefited population maintenance. In the present study, the overall female-to-male ratios were 1.19:1 for *C. zillii* and 2.01:1 for *O. niloticus*. Both populations showed a female-biased ratio. A high proportion of females enhanced population reproductive potential [37]; therefore, the high female-to-male ratio of tilapia in the Jinghong Reservoir likely facilitated the continued expansion of these populations.

The successful expansion of invasive fish was closely linked to the plasticity of their reproductive periods, as invasive species often exhibited flexible reproductive regulation [8]. Previous studies have shown that the breeding seasons of *C. zillii* and *O. niloticus* were highly variable in their native and invaded ranges. For example, in the native range in Egypt, the spawning periods for *C. zillii* in the Nile tributaries and Lake Timsah were from May to September and from January to August, respectively [38,39]; in invaded ranges, its breeding season varied more widely, with some populations breeding year-round and others only from June to August [40,41]. Similarly, for *O. niloticus* in its native range, some populations breed year-round [42], while others breed from February to September or May to December [43]; in invaded ranges, year-round breeding is more common [44,45]. In this study, the breeding seasons of *C. zillii* and *O. niloticus* in the Jinghong Reservoir were from April to September and from May to November, respectively, which differ somewhat from those in both their native and other invaded ranges. It was generally accepted that variations in breeding season were primarily influenced by environmental characteristics, with water temperature being a key factor [46]. Siddiqui [47] noted that *C. zillii* could spawn year-round in tropical regions. He et al. [48] found that tilapia in subtropical regions spawned only in specific months when temperatures were suitable. A study on tilapia in Rondegat River found that they spawned frequently when water temperatures were suitable [49]. The Jinghong Reservoir is located in a transitional zone between tropical and subtropical climates, with water temperatures remaining above 20 °C for most of the year, which falls within the suitable range for tilapia spawning. This condition created favorable conditions for population establishment and successful invasion of both species.

In this study, both absolute and relative fecundities of *C. zillii* were significantly higher than those of *O. niloticus*. This difference might be related to their spawning characteristics. *C. zillii* is a substrate-nesting spawner (non-mouthbrooder) [48], whereas *O. niloticus* is a mouthbrooding species [13]. *C. zillii* was smaller and generally less tolerant of environmental fluctuations, so its higher fecundity helped maintain a large population size. In contrast, mouthbrooding enhanced juvenile survival rates, but it typically resulted in lower fecundity compared to the non-mouthbrooding strategy [50].

From an invasion history perspective, surveys conducted by Zhang et al. [51] (2006–2015) in the Jinghong Reservoir did not record any tilapia. By 2019, Liu et al. [52] found that *O. niloticus* had invaded, but *C. zillii* went detected. Their results indicated that *O. niloticus* invaded the Jinghong Reservoir earlier. However, our fish survey from 2023 to 2025 revealed that the two tilapia species accounted for a significant proportion of the catch in terms of both abundance (28.45%) and biomass (50.10%), with *C. zillii* having a higher abundance than *O. niloticus*. These results suggested that although *C. zillii* invaded later, it possessed greater fecundity and population expansion capacity in the Jinghong Reservoir.

### 4.2. Interspecific Divergence in Reproductive Strategies and Coexistence

Divergence in reproductive strategies was an important pathway promoting the coexistence of species in the same habitat [53]. Traditionally, r-selected species were characterized by high fecundity, small body size and small egg diameter, whereas K-selected species exhibited low fecundity, large body size and large egg diameter [54]. Based on their tendency toward r- or K-selection, species could be further classified into r-biased and K-biased types [55]. In this study, the fecundity of *C. zillii* was significantly higher than that of *O. niloticus*, whereas its body length, body weight, size at first sexual maturity and egg diameter were significantly smaller. Based on these traits, *C. zillii* could be considered a more r-selected strategy, while *O. niloticus* exhibited a more K-selected strategy, indicating clear divergence in their reproductive strategies. This divergence reduced interspecific competition and promoted niche partitioning and coexistence of the two species in the Jinghong Reservoir.

The two tilapia species also exhibited distinctly different breeding seasons. *C. zillii* bred from April to September, peaking from May to June, while *O. niloticus* bred from May to November, peaking in August. This temporal staggering of breeding periods effectively reduced direct competition for reproductive resources between the two species, facilitating their coexistence and the formation of dominant populations in the Jinghong Reservoir. Similar phenomena have been observed between *C. zillii* and *Sarotherodon galilaeus* in the Shanmei Reservoir [35], as well as between *Rhinogobius cliffordpopei* and *R. giurinus* in Lake Erhai in China [19].

### 4.3. Management Strategies

In light of the invasion and coexistence patterns of the two tilapia species in the Jinghong Reservoir, the following management measures are recommended: (1) Strengthen supervision of aquaculture escapes and anthropogenic releases to minimize the risk of new introductions. (2) Intensively harvest breeding populations during the peak breeding seasons of *C. zillii* (May-June) and *O. niloticus* (August). This would enhance fishery utilization of these species, while providing a direct and effective means of controlling their population abundance. (3) Prioritize *C. zillii* for containment; given its higher fecundity and more rapid population expansion compared to *O. niloticus*, *C. zillii* should be designated as a high-priority management species. Strict monitoring and containment measures must be implemented to prevent its further dispersal into the upstream reaches of the Lancang River.

## 5. Conclusions

Both *C. zillii* and *O. niloticus* in the Jinghong Reservoir exhibited reproductive traits conducive to invasion, including early sexual maturation, prolonged breeding seasons and batch spawning. The two species showed clear divergence in their reproductive strategies: *C. zillii* adopted a more r-selected strategy (high fecundity, small body size, and small egg diameter), whereas *O. niloticus* exhibited a more K-selected strategy (lower fecundity, relatively larger body size, and relatively larger egg diameter). The temporal staggering of their breeding seasons effectively reduced direct competition for reproductive resources. Divergence in reproductive strategies was one of the important ecological mechanisms underlying the coexistence of the two tilapia species in the Jinghong Reservoir. This study provided scientific insights into the roles of different reproductive strategies in the invasion and coexistence of introduced fishes and established a scientific foundation for risk assessment and targeted control of non-native fishes in the Lancang River.

## Figures and Tables

**Table 1:** The body length and weight of *C. zillii* and *O. niloticus*.

Species	Total		Female		Male	
	Mean Body Length (mm)	Mean Body Weight (g)	Mean Body Length (mm)	Mean Body Weight (g)	Mean Body Length (mm)	Mean Body Weight (g)
*C. zillii*	143.0 ± 27.0 (range: 52.0–213.0)	134.9 ± 62.1 (range: 54.0–399.4)	144.0 ± 24.0 (range: 53.0–200.0)	135.1 ± 56.2 (range: 6.4–388.7)	146.0 ± 25.0 (range: 52.0–213.0)	143.0 ± 62.7 (range: 5.4–399.4)
*O. niloticus*	174.0 ± 33.0 (range: 120.0–280.0)	209.4 ± 97.5 (range: 67.3–815.0)	183.0 ± 25.0 (range: 127.0–280.0)	229.5 ± 87.6 (range: 74.3–815.0)	164.0 ± 27.0 (range: 120.0–250.0)	186.2 ± 96.4 (range: 67.3–591.5)

**Table 2:** Sex ratio of *C. zillii* and *O. niloticus* in different months.

Month	*C. zillii*			*O. niloticus*		
	Sex Ratio (F:M)	χ^2^ Value	*p* Value	Sex Ratio (F:M)	χ^2^ Value	*p* Value
January 2025	0.68	2.250	*p* = 0.134	1.38	1.754	*p* = 0.185
February 2025	1.27	0.610	*p* = 0.435	5.4	15.125	*p* < 0.01
March 2025	1.07	0.034	*p* = 0.853	5.29	20.455	*p* < 0.01
April 2025	1.29	0.500	*p* = 0.480	0.87	0.133	*p* = 0.715
May 2025	0.72	0.806	*p* = 0.369	1.5	1.200	*p* = 0.273
June 2025	1.31	0.533	*p* = 0.465	1	0.000	*p* = 1.000
July 2025	0.76	0.533	*p* = 0.466	2.3	5.121	*p* < 0.05
August 2025	1.50	2.174	*p* = 0.141	3.88	13.564	*p* < 0.01
September 2025	1.23	0.421	*p* = 0.516	2.55	7.410	*p* < 0.01
October 2025	1.19	0.348	*p* = 0.555	4.22	17.894	*p* < 0.01
November 2025	1.25	0.444	*p* = 0.505	1.33	0.714	*p* = 0.398
December 2025	1.21	0.959	*p* = 0.515	1.44	1.984	*p* = 0.159

**Table 3:** The fecundity indices of *C. zillii* and *O. niloticus*.

Species	Body Length (mm)	Body Weight	Sample Number	F (Eggs)		FL (Eggs/mm)		Fw (Eggs/g)	
				Range	Mean ± SD	Range	Mean ± SD	Range	Mean ± SD
*C. zilli*	110–200	50–400	46	2613.17–12,876.00	5678.39 ± 2006.83	18.40–67.53	38.18 ± 11.50	25.80–93.81	48.67 ± 15.80
*O. niloticus*	160–230	160–450	50	1037.00–2970.00	1742.52 ± 553.81	5.67–14.88	9.22 ± 2.50	4.25–15.40	8.18 ± 2.54

*F* is the individual absolute fecundity (number of eggs); *F_L_* is the length-relative fecundity (eggs/mm); *F_W_* is the weight-relative fecundity (eggs/g).

## Data Availability

Data are contained within the article.
